# Instrumentation and control in a laboratory-scale process for CO_2_ capture post-combustion

**DOI:** 10.1016/j.ohx.2026.e00817

**Published:** 2026-07-26

**Authors:** María Fernanda Silva León, Erick Omar Castañeda Magadán, Miriam Navarrete Procopio, Víctor Manuel Zezatti Flores, Ángel Tlatelpa Becerro

**Affiliations:** a Facultad de Ciencias Químicas e Ingeniería, Universidad Autónoma del Estado de Morelos (UAEM), Av. Universidad 1001, Col. Chamilpa, C.P. 62209 Cuernavaca, Morelos, Mexico; b Centro de Investigación en Ingeniería y Ciencias Aplicadas, Universidad Autónoma del Estado de Morelos (UAEM), Av. Universidad 1001, Col. Chamilpa, C.P. 62209 Cuernavaca, Morelos, Mexico; c Escuela de Estudios Superiores de Yecapixtla, Universidad Autónoma del Estado de Morelos (UAEM), Avenida Félix Árias, C.P. 62824 Yecapixtla, Morelos, Mexico

**Keywords:** Volumetric flow control, Temperature acquisition, Low-cost acquisition data, Post-combustion process, CO_2_ capture

## Abstract

The present study shows the instrumentation and control of a laboratory-scale post-combustion CO_2_ capture process (CO_2_-CP), including the implementation of a low-cost data acquisition system for monitoring and controlling the key process variables. This system simulates the capture of flue gases generated in thermoelectric power plants, as well as the operation of packed columns commonly employed in large-scale industrial applications.

To ensure effective control, the developed platform continuously acquires and processes the operating variables throughout the experimental tests. Temperature and flow rate are acquired in real time using an open-source graphical user interface.

Once the monitoring system was validated, experimental tests were conducted to evaluate the response of the CO_2_ absorption process under operating conditions. The experiments were performed using a 10% monoethanolamine (MEA) solution, where the absorption process exhibited an exothermic reaction, producing an approximately 2 °C temperature increase at the outlet of the absorption column and reaching saturation after approximately 300 s.

In addition to monitoring the process’s thermal behavior, maintaining mass balance throughout the system is essential to ensure stable operation and compliance with the continuity principle, whereby the inlet flow rate equals the outlet flow rate, thereby contributing to process stability and measurement reliability.

## Graphical abstract

**Figure:**
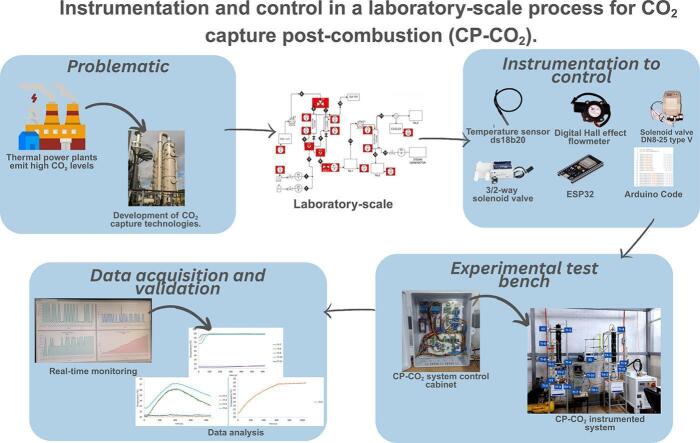

## Specifications table

**Table:** 

Hardware name	*Low-cost DAQ for a post-combustion process.*
Subject area	• Electrical − electronic engineering • System data acquisition (DAQ) • Chemical engineering • Educational tools and open-source alternatives to existing infrastructure
Hardware type	• Measuring process variables and in-lab sensors • Field measurements and sensors • Electrical engineering and computer science
Closest commercial analog	• Development boards (Arduino, Blue Pill, Raspberry Pi) • Programmable Logic Controller • National Instruments • Agilent Technology.
Open source license	Creative Commons Attribution 4.0 International (CC BY 4.0)
Cost of hardware	*USD 355.5*
Source file repository	Zenodo [https://doi.org/10.5281/zenodo.20735751]

## 1 Hardware in context

Thermal power plants are the main emitters of CO_2_, with a significant carbon footprint estimated at 6.52 million tons. Of this large volume of emissions, 90.23% comes directly from the burning of fossil fuels such as coal, oil, and natural gas, contributing significantly to total greenhouse gas emissions. As a result, these emissions directly contribute to global climate change, which exacerbates phenomena such as rising Earth's average temperature and altered weather patterns [2], [3].

To mitigate these environmental impacts, considerable research has focused on developing carbon capture technologies to reduce CO_2_ emissions from industrial sources. A CO_2_-CP employs amine-based solvents to mitigate CO_2_ emissions from thermal power plants [4], [5], [6]. The core components of the post-combustion capture and regeneration process are packed columns. An absorption column with specific dimensions can be filled randomly; conversely, a packed desorption column is utilized to regenerate the absorbent solution [7].

Due to the efficiency of the absorption and regeneration processes strongly depends on stable operating conditions, accurate instrumentation and process control become essential. Chemical process instrumentation and control emphasize the automation of variables such as temperature, flow, and chemical composition to ensure process stability and safety. Likewise, in CO_2_-CP, instrumentation and control aim to optimize operational efficiency, manage changes in operating conditions, and lower operating costs through advanced control strategies [8], [9], [10], [11]. The implementation of temperature acquisition and control ensures stability within fixed limits, maintains consistent conditions for different processes, and improves efficiency. [12].

At the industrial level, these monitoring and control tasks are commonly performed using dedicated automation platforms specifically designed for continuous process operation. Pilot plants for the CO_2_ capture process (CO_2_-CP) typically manage operations using a programmable logic controller (PLC) or a distributed control system (DCS), both connected via Ethernet or Wi-Fi to a manufacturer-specific processor card linked to input/output modules, while large-scale capture plants require advanced control systems to promptly respond to load variations and minimize the time needed to return to steady-state operating conditions. [13], [14]. These cards connect directly to individual devices, such as temperature sensors, pressure transmitters, analyzers, flow transmitters, and control valves, for devices that operate with a 4–20 mA signal [15]. The choice between a PLC and DCS is influenced by the installation cost. It may be more practical to use one if only a small number of devices are needed, while the other may be more feasible or even preferable for larger installations [16].

Despite the complex of industrial automation systems, their high acquisition and maintenance costs often limit their implementation in laboratory-scale research facilities. In experimental process instrumentation, low-cost systems have emerged as effective and accessible alternatives for data acquisition and monitoring of key variables. This approach enables the replacement of expensive industrial equipment—such as CompactRIO modules or proprietary SCADA systems—with open-hardware platforms that provide sufficient accuracy and flexibility for academic and applied research purposes. [17].

Consequently, the adoption of low-cost instrumentation has become increasingly attractive for experimental research and engineering education. Laboratory-scale instrumentation, control, and automation are crucial to understanding thermal, mechanical, hydrological, and chemical processes, contributing to model predictions, as well as developing reliable tests, and reducing manual intervention [18], [19], [20].

In this context, the present work proposes a low-cost instrumentation and control platform for a laboratory-scale CO_2_ capture process, offering both practical and scientific contributions. The main contributions of this work are:

Prediction of MEA saturation time during experimental tests under different solvent concentrations.

Real time monitoring of the saturation curve during the CO_2_ capture process.

The visibility of these types of processes at laboratory scale, and the importance of students having equipment instrumented and controlled in such a way that there is the least possible intervention, to obtain reliable data, since there is currently no literature on the instrumentation and control of such processes at laboratory scale.

Implementation of a low-cost, real-time data acquisition system, based on calibrated sensors, enabling reliable measurements and providing high-quality datasets for subsequent data analysis and process optimization.

To highlight the advantages of the proposed platform, Table 1 presents a comparison with another system implemented in the industrial sector:

**Table 1:** Comparative system.

Variable	Industrial implementation	CO_2_-CP System	
Temperature	Resistance temperature detectors (RTDs), such as Pt100 and Pt1000, are known for their high accuracy but are expensive and require complex networking. [21]	DS18B20 digital sensors (±0.5 °C) installed at strategic points in the system	The data acquired from the CO_2_-CP System show it is working correctly, making it an excellent option for demonstrating the use of these technologies on a laboratory scale.
Flow	Certified industrial flowmeters with low latency. [14]	Flowmeter Hall effect digital flowmeters, which operates measuring flow in the range of 50 to 800 mL/min.	The installed Hall effect flowmeter allows liquid measurements in the laboratory, demonstrating the correct operation of the system.

## 2 Hardware description

**Fig. 1:**
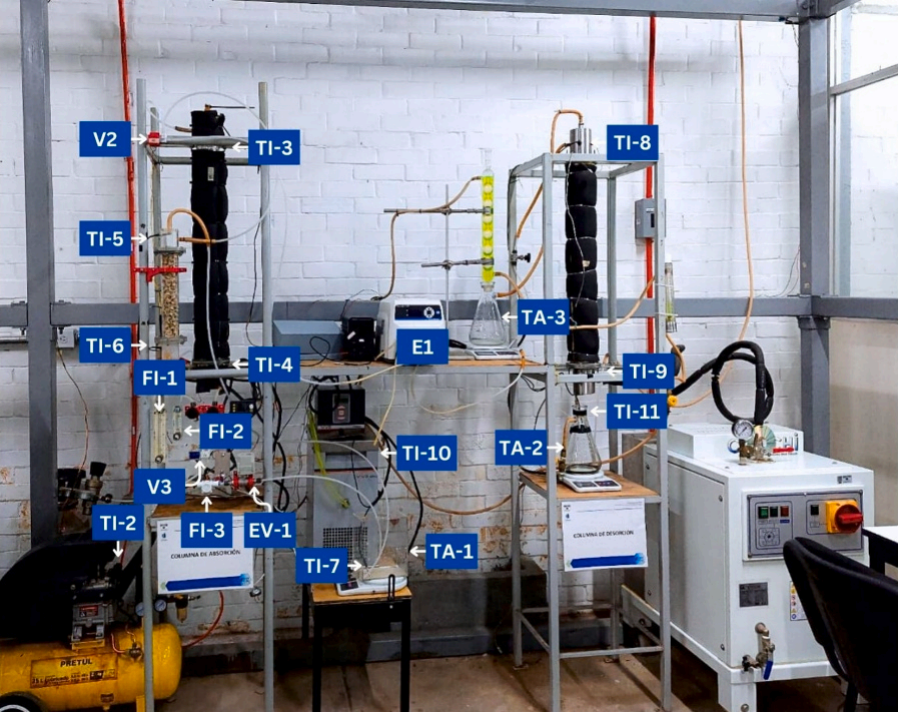
Complete system with monitoring points.

The CO_2_-CP requires the instrumentation and control of several key variables for the study in question. Due to the number of sensors and control instruments, it was necessary to design and manufacture specific electronic boards to connect the sensors, to obtain a stable system during data acquisition and processing of the received signals. Fig. 1 shows the real complete system, highlighting the monitoring points of interest, as well as the location of the sensors and control instruments.

For monitoring the CO_2_-CP, the following variables were instrumented: temperature, volumetric flow, and mass. Table 2 shows the nomenclature of the sensors instrumented in the process.

**Table 2:** Sensor nomenclature.

Nomenclature Sensor	Description
TI-1	Solution inlet temperature.
TI-2	Ambient temperature.
TI-3	Gas outlet temperature (0.70 m packed area).
TI-4	Solution outlet temperature (0.70 m packed area).
TI-5	Gas outlet temperature (0.25 m packed area).
TI-6	Solution outlet temperature (0.25 m packed area).
TI-7	Saturated solution temperature.
TI-8	Steam outlet temperature.
TI-9	Steam inlet temperature.
TI-10	Cooling fluid temperature.
TI-11	Regenerated solution outlet temperature.
FI-3	Digital hall effect flowmeter.
EV-1	Proportional solenoid valve.
V1	3/2-way valve for airflow direction (inlet).
V2	3/2-way valve for solution flow direction (inlet).
V3	3/2-way valve for solution flow direction (outlet).

### 2.1 Basic function of the temperature sensor

**Fig. 2:**
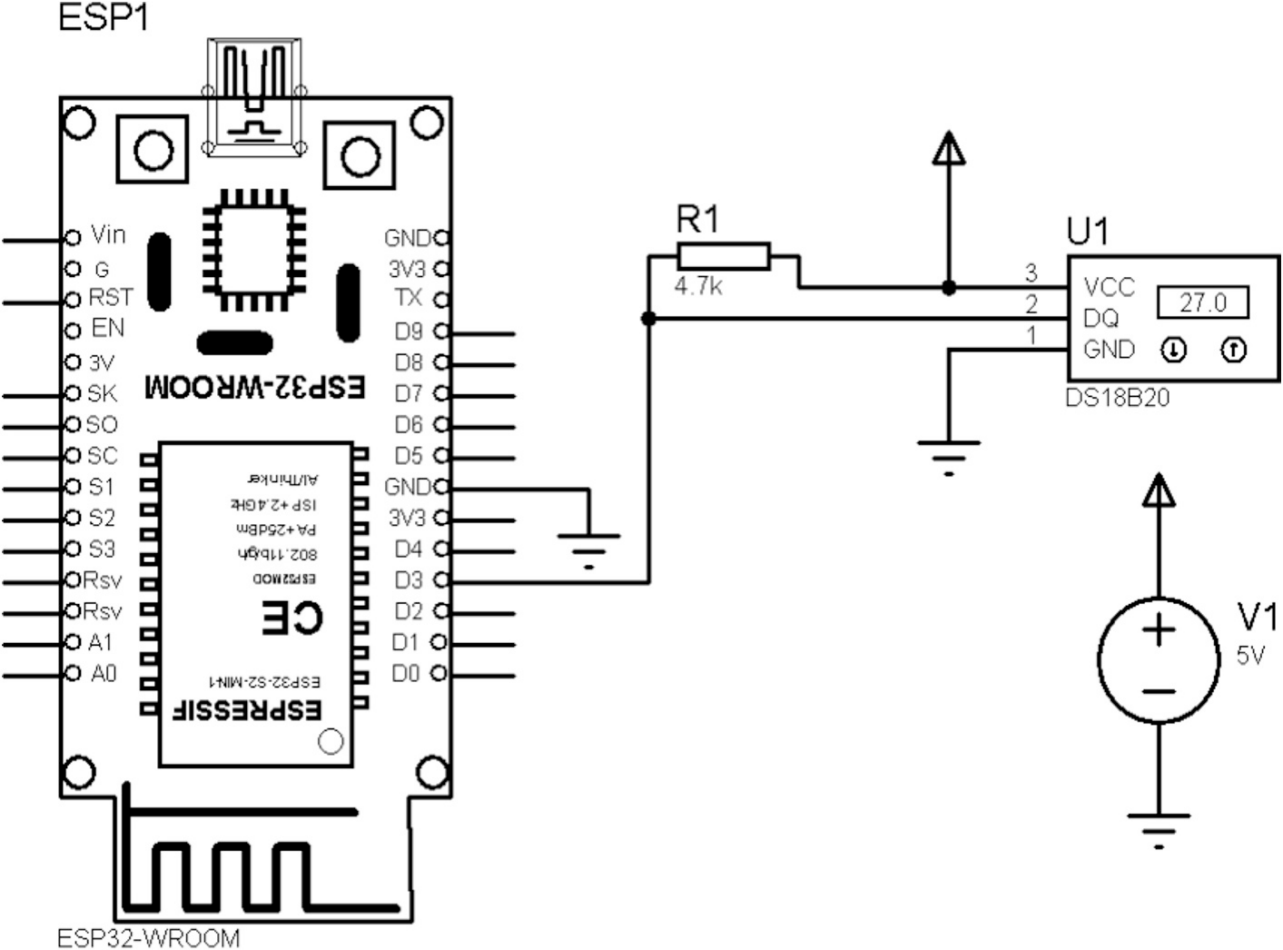
The electrical connection of the temperature sensor.

For temperature monitoring at various points of interest, DS18B20-type digital temperature sensors were utilized. These sensors operate with a 5 V DC power supply and are waterproof, featuring a stainless-steel cover that encases their outer tip. Their operational range is −55 °C to + 125 °C, with an accuracy of ± 0.5 °C between −10 °C and + 85 °C and a configurable resolution of 9 to 12 bits. Additionally, they communicate using a single pin. The DS18B20 sensor was selected and implemented since the measurement intervals are within the appropriate temperatures for the currents in CO_2_-CP, such as the MEA, in the experimental tests the highest obtained temperature is approximately 27 °C. Furthermore, the highest value of saturated steam is approximately 95 °C. The electrical connection diagram for the digital temperature sensor is illustrated in Fig. 2.

### 2.2 Basic function of the flowmeter

For volumetric flow control of the absorbed solution, a commercial Hall effect digital flowmeter was used, which operates with an input voltage of 5 V DC and allows measuring flow in the range of 50 to 800 mL/min.

**Fig. 3:**
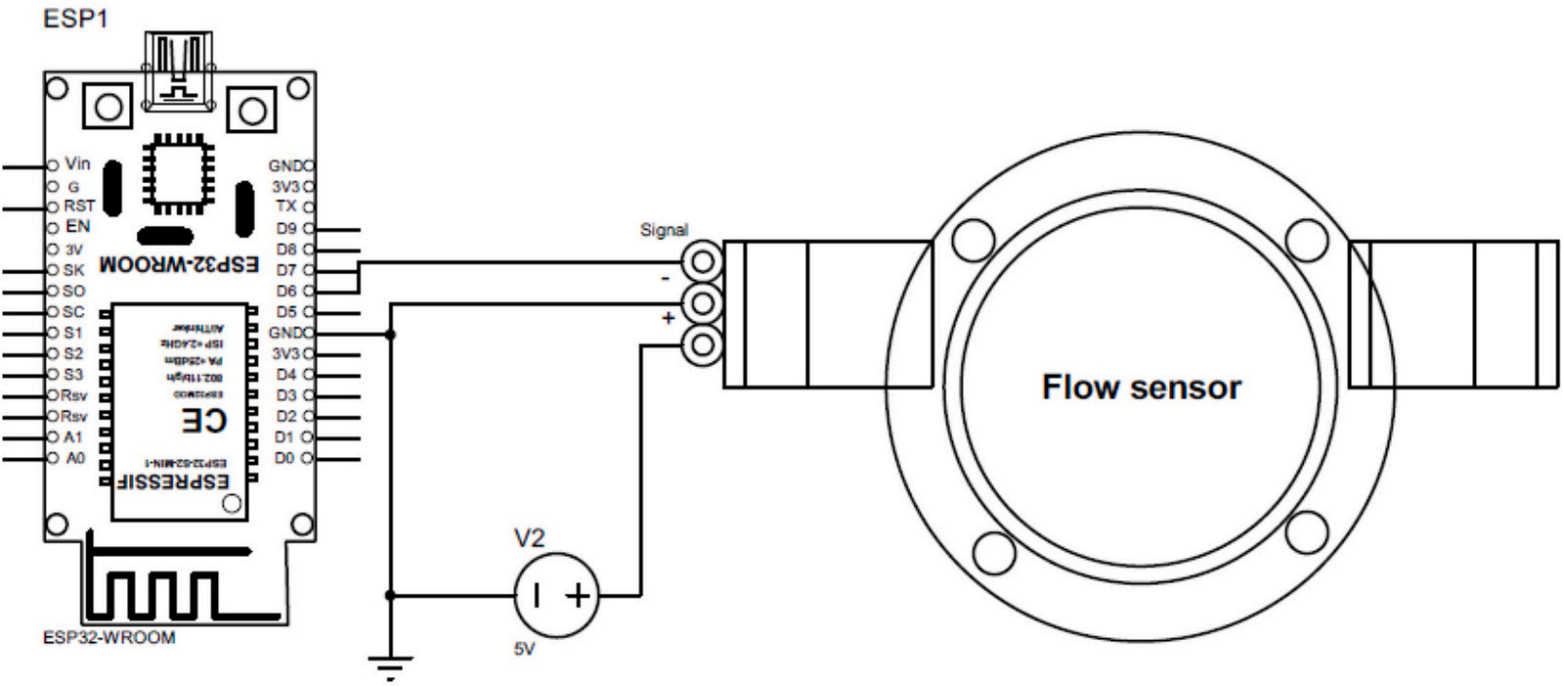
Electrical connection of the flowmeter.

**Fig. 4:**
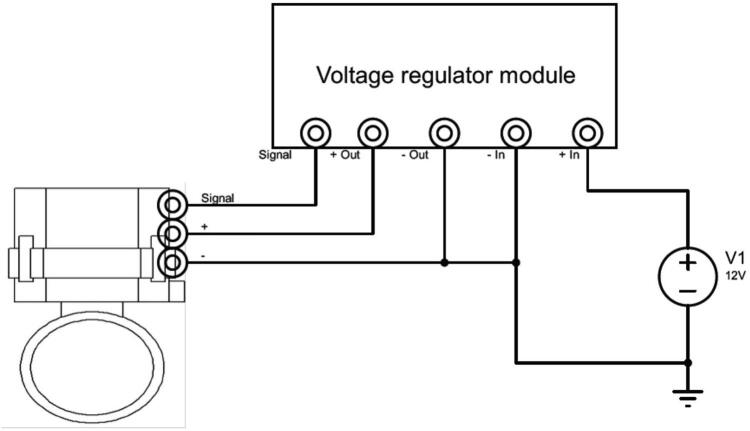
Electrical connection of the flow control system.

This flowmeter works with small flows that occur in the system, which are favorable for the absorption column since the flows it handles are within the range of this component. In addition, a DN8-25 type V proportional ball solenoid valve was implemented, which operates with a voltage of 0 to 10 V DC. The opening and closing of the solenoid valve are controlled by a voltage regulator module, which adjusts the necessary voltage according to the degree of opening required to achieve the volumetric flow defined by the user. Fig. 3, Fig. 4 show the electrical diagrams of the digital flowmeter, the proportional solenoid valve, and the voltage regulator module used in the system.

### 2.3 Basic function of the 3/2-way solenoid valve

**Fig. 5:**
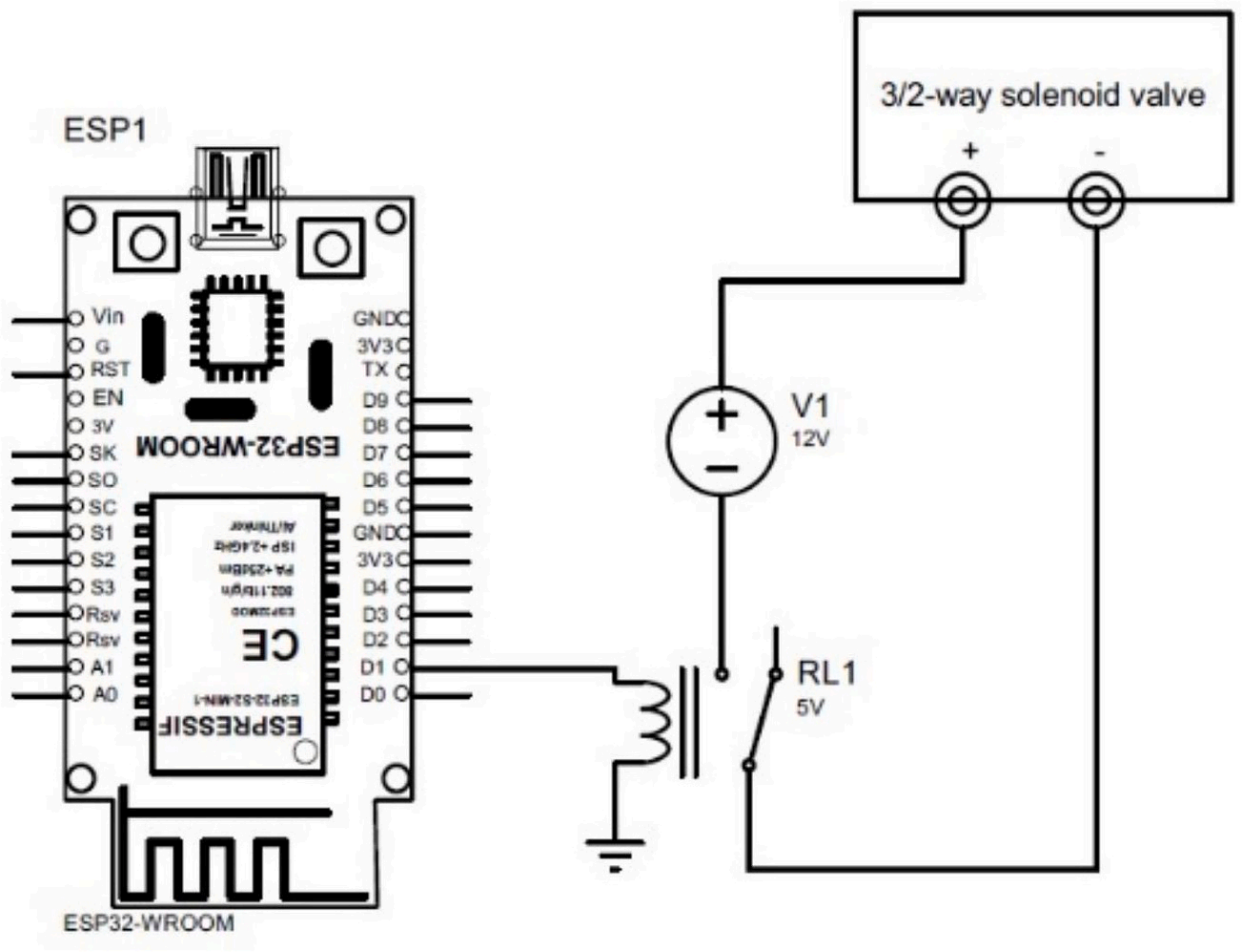
Electrical connection of the 3/2-way solenoid valve.

The post-combustion CO_2_ capture process employs three 3/2-way solenoid valves that operate at a voltage of 12 V DC together with a relay module that operates at 5 V DC. The valve system allows the flow of air current and absorbent solution to be directed between the two columns according to the configuration desired by the user. These valves are located at the inlets and outlets of the columns to direct the currents of Air, CO_2_, Monoethanolamine (MEA), and saturated MEA. Fig. 5 shows the electrical connection diagram of the solenoid valves together with the relay modules.

## 3 Design files summary

All designs are available in an editable format and archived in the Zenodo repository [1]. It shows all the documentation and designs implemented in the CO_2_-CP system. Table 3 shows a Design Files Summary.

**Table 3:** Design Files Summary CO_2_-CP system.

File	Description	Section
temperature.ino	Arduino code of one temperature sensor	2.1
temperature_ds18b20_datasheet.pdf	Temperature sensor datasheet	2.1
flowmeter_proteus.pdsprj	Electrical connection of the flowmeter	2.2
volumetric_flow_code.ino	Arduino code of the flowmeter	2.2
Voltage regulator module_datasheet.pdf	Voltage regulator module datasheet	2.2
stream__select_code.ino	Arduino code to control one solenoid valve	2.3
stream_select.pdsprj	Electrical connection of one solenoid valve	2.3
temperature_aborption.pdsprj	Electrical connection of absorption temperature sensors	5.3
temperature_desorption.pdsprj	Electrical connection of desorption temperature sensors	5.3
temperature_system_code.ino	Arduino code of both columns’ temperature sensors	5.3
stream_select_system_code.ino	Arduino code to control three solenoid valves	5.6
stream_select_system_simulation.pdsprj	Electrical connection of three solenoid valve	5.6
*PCB_1.cvr3d*	PCB 1 Aspire Files	5.7
PCB_2.cvr3d	PCB 2 Aspire Files	5.7
*PCB_1.gcode*	PCB 1 G code to manufacture	5.7
PCB_2.gcode	PCB 2 G code to manufacture	5.7
*PCB_1.pcb*	PCB 1 Printed Circuit Board files	5.7
PCB_2.pcb	PCB 2 Printed Circuit Board files	5.7
CO2-CP system.pdf	Operating manual system	6

## 4 Bill of materials summary

Table 4 shows the necessary elements for the control and acquisition system of the post-combustion CO_2_ capture process variables at the laboratory scale. It also shows the electronic components and total cost of the CO_2_-CP system.

**Table 4:** Bill of materials and costs for CO_2_-CP system implementation.

Component	Model	Employed elements	Cost per unit −currency	Total cost −currency	Source of materials	Code	Operating interval
Microprocessor	ESP32 NodeMCU-32S	2	USD 9	USD 18	Amazon	NA	5 V DC
Temperature sensor	DS18B20	11	USD 3	USD 33	Amazon	TI	−55° C − 125° C
Power supply	NANKADF	1	USD 80	USD 80	Amazon	NA	
Hall Effect Flowmeter	sensor TDS	1	USD 13.5	USD 13.5	Amazon	FI	50 – 800 mL/min
Voltage regulator module	DC Buck-Boost Converter	1	USD 46	USD 46	Amazon	NA	0 – 32 V DC
Solenoid valve	DN8-25 V-type ball solenoid valve	1	USD 75	USD 75	AliExpress	EV	0 – 10 V DC
3/2-way solenoid valve	2-position 3-way mini solenoid valve.	3	USD 30	USD 90	Amazon	V	12 V DC
Relay Module	2-channel relay module.	2	USD 6	USD 12	Amazon	NA	5 V DC
Resistances	Electrical resistance, 4.7kΩ	11	USD 18	USD 18	Amazon	NA	NA

## 5 Build instructions

### 5.1 Absorption and desorption process

This section presents the electrical connection diagrams for each process variable, implementing the components listed in Table 4.

**Fig. 6:**
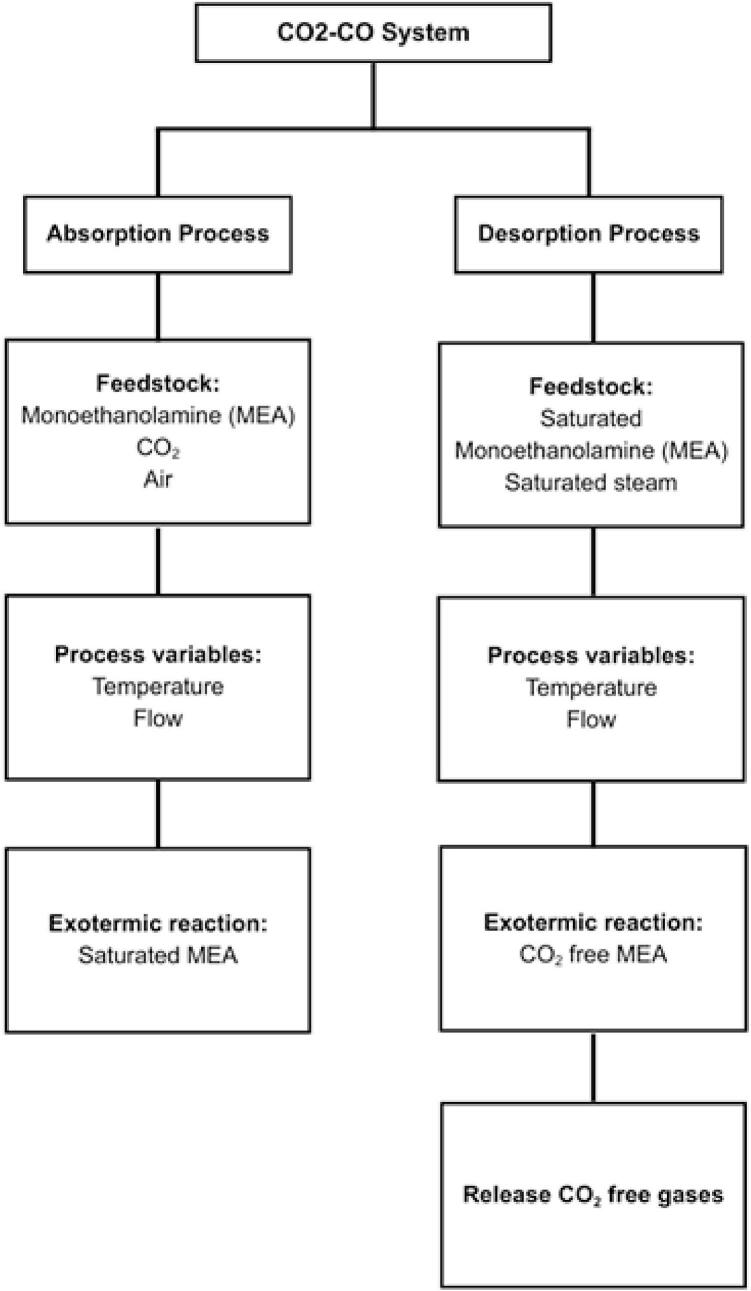
Block diagram for CO_2_ −CP system description.

The CO_2_-CP system consists of two main processes: absorption and desorption. Each process is represented by a block diagram, as shown in Fig. 6. The absorption process uses the following raw materials: monoethanolamine (MEA), the chemical absorbent; CO_2_, the solute to be absorbed from the gas stream; and air, used to dilute the solute to the required concentration for the experiment.

The desorption process uses a monoethanolamine solution saturated with CO_2_ and saturated steam, which serve as inert gases to desorb the CO_2_ from the solution. The process variables monitored and controlled in both processes are temperature and volumetric flow rate. Finally, during absorption, MEA absorbs CO_2_ via an exothermic reaction, releasing energy and heating the absorbent solution. This process produces a saturated MEA solution containing the absorbed CO_2_. In the desorption process, the CO_2_ is separated from the saturated MEA to regenerate the absorbent and release the gas. This process releases CO_2_ as a gas, and the regenerated MEA is recirculated back into the absorption process.

### 5.2 Description process CO_2_-CP

**Fig. 7:**
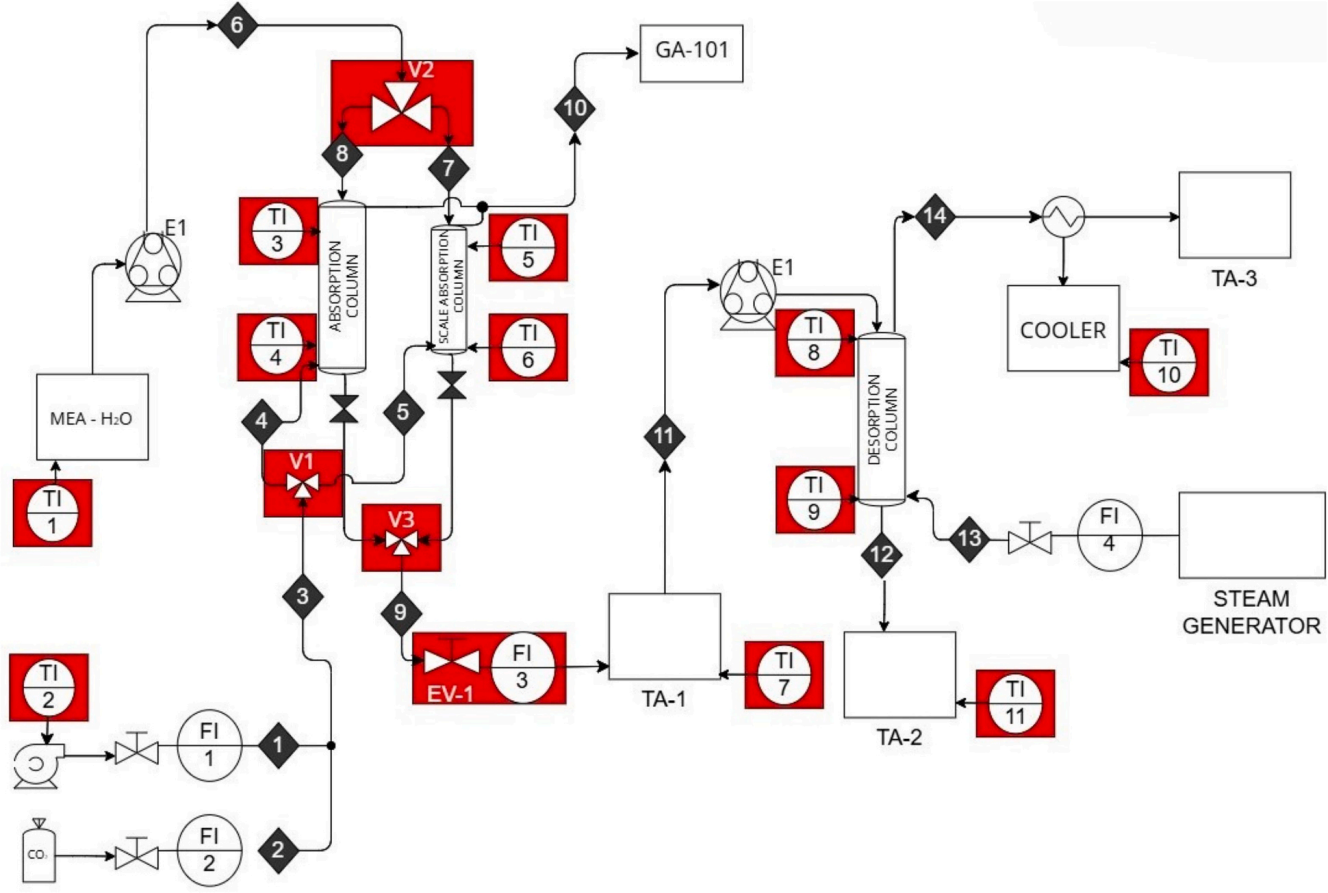
Post-combustion process on a laboratory scale diagram.

The CO_2_-CP at a laboratory scale, situated in the Separation Processes Laboratory of the Unitary Operation Laboratory (LOU-FCQeI) at the Universidad Autónoma del Estado de Morelos (UAEM), is divided into two operational units: an absorption unit and a desorption unit. The CO_2_-CP comprises two packed columns; one is 0.076 m (3 in) in diameter and 0.70 m in length, made of 304 stainless steels, while the other column is 0.042 m (2 in) in diameter and 0.25 m in length, constructed from acrylic for the absorption process. Each column features two inlets and two outlets: one inlet at the top (liquid inlet) and one at the bottom (gas inlet), with one outlet located at the top (gas outlet) and another at the bottom (liquid outlet). Fig. 7 illustrates the complete system using a piping and instrumentation diagram (P&ID).

The capture process starts at the bottom of the absorption columns, which are fed by a gas mixture of CO_2_-Air (streams 1 and 2) coming from an air compressor and a CO_2_ storage tank, passing through two flowmeters (Fl-1, Fl-2). The gas mixture enters a 3/2-way valve (V1) located at the bottom of the columns (stream 3). This valve directs the gas mixture towards a larger capacity absorption column (1600 mL) or a smaller capacity one (125 mL), streams 4 and 5 correspondingly. An absorbent solution located in a storage tank (MEA-H_2_O) is fed through the top of the absorption columns (stream 6) employing a peristaltic pump (E1). The supplied absorbent solution reaches a 3/2-way valve (V2), which allows the filling of the 1600 mL or 125 mL column (streams 7 and 8). The absorbent solution is put in countercurrent contact with the CO_2_-Air mixture through random packings, generating an exothermic reaction and increasing the temperature of the solution at the outlet. The CO_2_-saturated absorbent solution gets out of the bottom of the operating absorption column (stream 9) through a 3/2-way valve (V3). The saturated CO_2_ absorbent solution is released through stream 9, passing through a digital Hall effect flowmeter (FI-3) and a proportional solenoid valve (EV-1), arriving at a storage tank (TA-1). The CO_2_-free gas stream gets out to the top of the absorption column until it reaches a gas analyzer (stream 10). From the storage tank TA-1, the saturated solution obtained from the absorption process is pumped through the peristaltic pump (E1) to the desorption column (stream 11). Saturated steam (stream 13) from a steam generator enters the bottom of the desorption column, creating countercurrent contact between the fluids in the packed desorption column to regenerate the solution. After desorption, the regenerated solution is out (stream 12) at the bottom of the column and is stored in a storage tank (TA-2). Finally, water vapor and CO_2_ 6 get out of the top of the column, passing through a heat exchanger. The condensate (stream 14) is stored in a storage tank (TA-3).

For the development of the research work, the post-combustion CO_2_ capture system was fully implemented: a digital micro flowmeter by Hall effect (FI-3), a proportional solenoid valve (EV-1), a voltage regulator module, and 3 3/2-way solenoid valves at the inlet and outlet of the absorption and desorption columns respectively (V1, V2, V3). In addition, to monitor the temperature variable, the system was equipped with a total of 11 temperature sensors; two of them at the top and bottom of the 1600 mL absorption column (TI-3, TI-4), two at the top and bottom of the 125 mL absorption column (TI-5, TI-6), a sensor immersed in the absorbent solution storage tank (TI-1), one exposed to the environment (TI-2), one in the storage tank (TA-1) of the saturated absorbent solution (TI-7), two sensors at the top and bottom of the desorption column (TI-8, TI-9), a sensor located in the heat exchanger (TI-10) and another in the storage tank (TA-4) submerged in the regenerated solution (TI-11).

### 5.3 Temperature

**Fig. 8:**
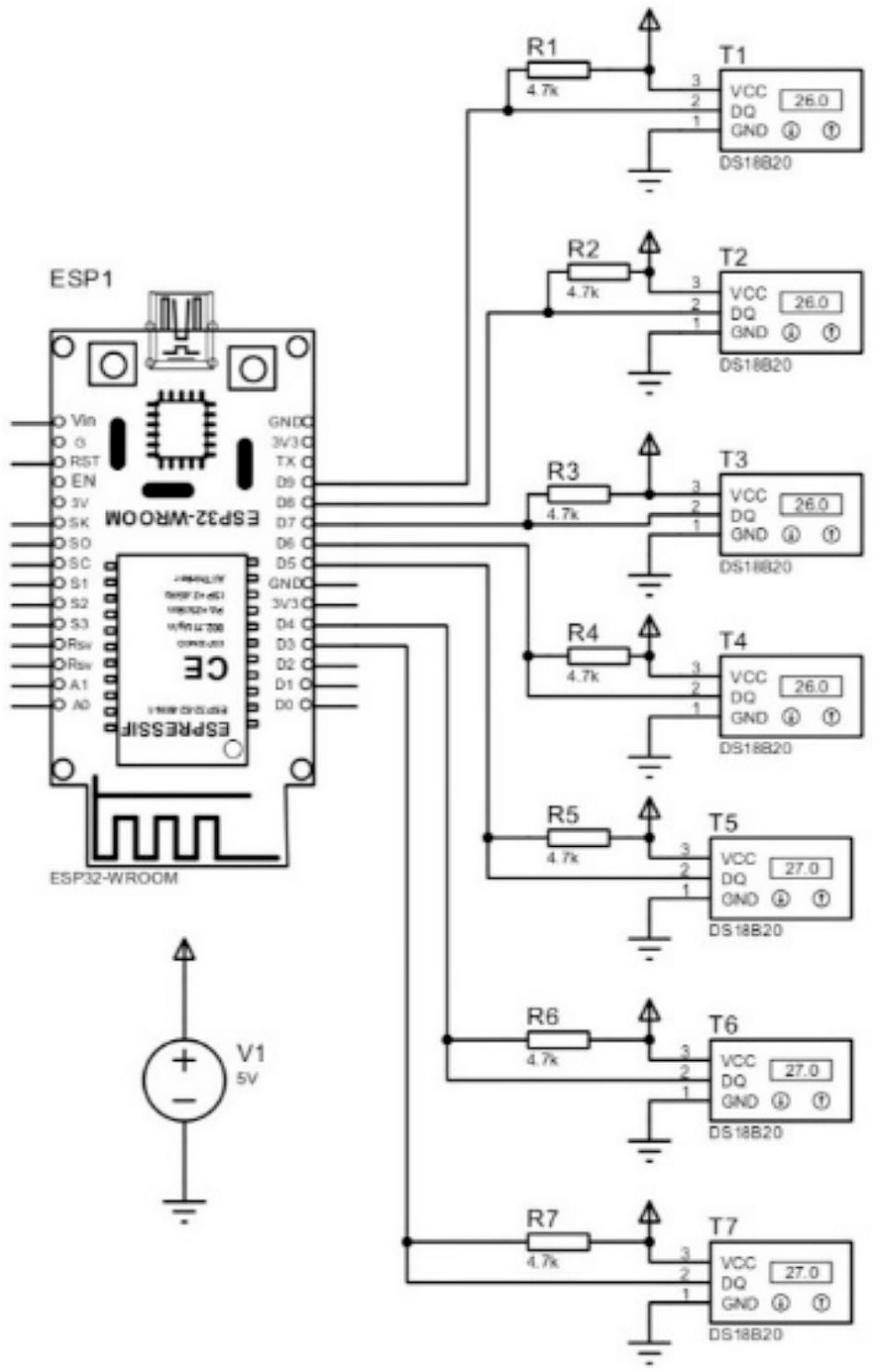
Electrical connection temperature sensor for the desorption column.

**Fig. 9:**
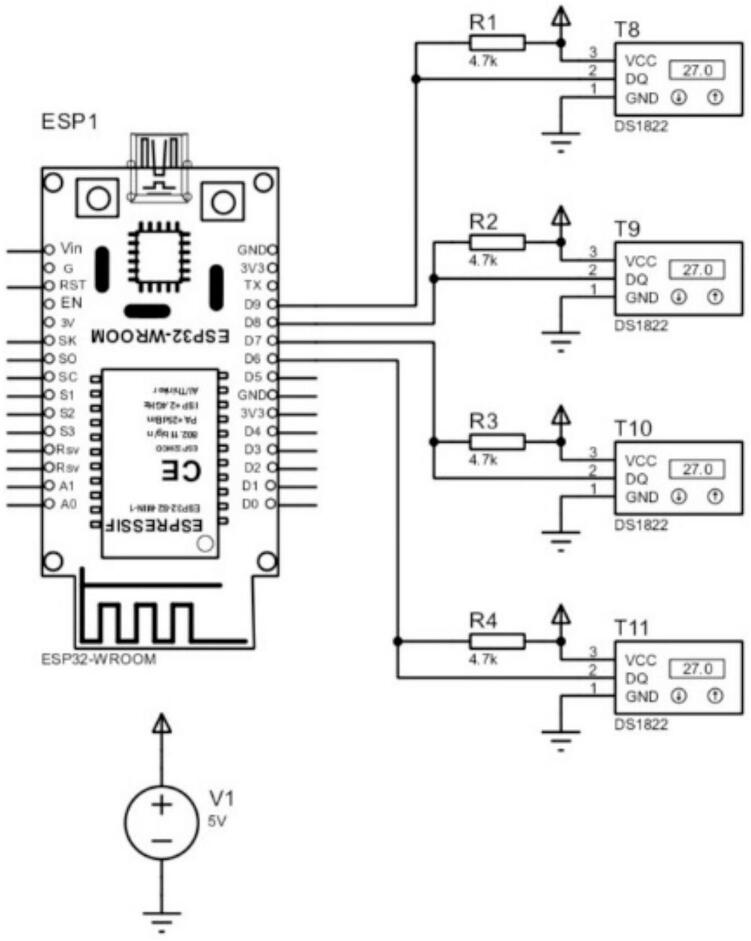
Electrical connection of temperature sensor for absorption columns.

Eleven temperature sensors were implemented at different points of interest in the post-combustion process for data acquisition. The sensors are divided into two phenolic plates, one for the absorption columns and one for the desorption column. Fig. 8 shows the electrical connections diagram of the 7 sensors located in the absorption columns, and Fig. 9 shows the electrical connections diagram of the 4 sensors located in the desorption column.

### 5.4 Process calibration

The calibration of the temperature sensors was performed using a thermal bath which contains a uniform fluid, and the temperature can be adjusted. The test was made at different temperatures which were compared with the temperature acquired by the CO_2_-CP system sensors, established by reference the thermal bath.

Once the sensors have been installed and connected, the data acquisition system is calibrated and adjusted. Calibration involves comparing measurements obtained from the instrument under evaluation with those from a reference standard to quantify and correct any deviations in the recorded values.

To perform this procedure, a thermal bath is employed, providing a controlled, uniform temperature environment that can be maintained at stable operating conditions across a range of setpoints. The sensors are first identified, then immersed in the thermal bath fluid to record measurements under each test condition.

The calibration process begins at 5 °C and continues in successive increments of 5 °C until reaching 115 °C. At each temperature setpoint, the sensor readings are recorded and compared with the reference values. This procedure enables the determination of the calibration and correction equations needed to improve measurement accuracy and reliability during experimental operation.

The coefficient of determination (R^2^) indicates how well the calibration equation represents the experimental data. Its value ranges from 0 to 1, with values closer to 1 indicating a better fit. R^2^ is calculated using Equation (1):

(1) $$R^{2}=1-\frac{\sum {\left(y_{i}-{\hat{y}}_{i}\right)}^{2}}{\sum {\left(y_{i}-\overset{¯}{y}\right)}^{2}}$$

where ($y_{i}$) is the reference temperature, (${\hat{y}}_{i}$) is the temperature estimated by the calibration equation, and ($\overset{¯}{y}$) is the mean value of the reference temperatures.

The Root Mean Square Error (RMSE) quantifies the standard deviation of the residual errors after applying the calibration correction. An RMSE value of approximately 0.05 °C is generally considered excellent; values around 0.20 °C are considered good, whereas values greater than 0.5 °C are relatively high for calibrated temperature sensors. RMSE is calculated using Equation (2):

(2) $$\mathrm{RMSE}=\sqrt{\frac{1}{n}\sum_{i=1}^{n}{\left(y_{i}-{\hat{y}}_{i}\right)}^{2}}$$

where the error term is defined by Equation (3):

(3) $$e_{i}=y_{i}-{\hat{y}}_{i}$$

The Mean Absolute Error (MAE) represents the average magnitude of the errors regardless of their sign. It provides a direct measure of the average deviation between the measured and reference values and is calculated using Equation (4):

(4) $$\mathrm{MAE}=\frac{1}{n}\sum_{i=1}^{n}\left|y_{i}-{\hat{y}}_{i}\right|$$

The Maximum Error corresponds to the largest absolute deviation recorded throughout the entire calibration procedure and is determined using Equation (5):

(5) $${\mathrm{Error}}_{\max}=max\left|e_{i}\right|$$

Finally, the Corrected Accuracy, which reflects the measurement accuracy after applying the calibration correction, is calculated using Equation (6):

(6) $$\mathrm{CA}=\left(100-MAE\right)*100\%$$

Table 5 shows the correction equations for the most interesting temperature sensor in the CO_2_-CP system.

**Table 5:** Correction equations of temperature sensor.

Sensor	Correction Equations	R^2^ (−)	RMSE (−)	MAE(°C)	Max AE (°C)	CA(%)
TI-3	TI3=0.9932TI1+1.0509	0.99764	0.00509	0.82931	1.024	95.25
TI-4	TI4=0.9897TI2+0.5870	0.99967	0.00180	0.25247	0.660	97.82
TI-5	TI5=0.8420TI5+1.3777	0.99723	0.00864	0.86575	1.377	94.34
TI-6	TI6=0.9932TI6+0.3547	0.99989	0.00052	0.14484	0.355	98.79
TI-7	TI7=0.9848TI7+1.0226	0.99761	0.00518	0.83491	1.035	95.21
TI-8	TI8=0.9693TI8+1.6035	0.99966	0.00178	0.25723	0.656	97.81
TI-9	TI9=0.9911TI9+0.7678	0.99722	0.00866	0.86850	1.379	94.33
TI-11	TI11=0.9938TI11+0.3525	0.99989	0.00055	0.14561	0.364	98.77

In the proposed monitoring and instrumentation system, the DS18B20 temperature sensor was selected due to its low cost, wide operating range, and ease of integration with the ESP32 microcontroller. This digital device uses a 1-Wire communication interface, allowing multiple sensors to be connected to a single data pin, thereby reducing wiring complexity and eliminating the need for signal conditioning circuits.

Unlike PT100 RTD sensors, which require analog compensation circuits and periodic calibration to ensure accuracy, the DS18B20 provides direct digital readings, simplifying data acquisition and improving the system’s modularity. Furthermore, its significantly lower cost makes it an ideal option for research, educational, or automation platforms focused on low-cost instrumentation, where affordability and simplicity are key design considerations.

Table 6 shows a comparative analysis between both sensors, highlighting the advantages of the DS18B20 in terms of cost, connection simplicity, and applicability in experimental processes instrumented through the ESP32 platform.

**Table 6:** Comparative temperature sensors.

Criterion	DS18B20 Sensor	PT100 (RTD) Sensor	Comments/Preference
Signal type	Digital (1-Wire)	Analog (resistance variation)	The DS18B20 eliminates the need for analog conditioning, simplifying the design.
Measurement range	–55 °C to + 125 °C	–200 °C to + 850 °C	The PT100 has a wider range, but the DS18B20 adequately covers most laboratory and experimental processes.
Resolution / Accuracy	12-bit (±0.5 °C typical)	High precision (±0.1 °C or better)	The PT100 provides higher accuracy, although the DS18B20 is sufficient for experimental and educational measurements.
Calibration required	No complex calibration required	Periodic calibration required	Advantage for the DS18B20 in educational and quick test systems.
Ease of connection	1-Wire digital interface (single data pin, multiple sensors on shared bus)	Requires Wheatstone bridge or signal conditioning circuit	The DS18B20 offers direct integration with microcontrollers (Arduino, ESP32).
Approximate cost	3–5 USD	25–40 USD (without conditioning)	Major advantage of the DS18B20 for low-cost instrumentation projects.
Power supply	3.0–5.5 VDC	Stable excitation current (100 μA–1 mA)	The DS18B20 is simpler in design and power requirements.
Communication / reading	Direct digital, 1-Wire protocol	Analog conversion using external ADC or conditioning module	The DS18B20 reduces the need for additional hardware.
Typical applications	Thermal monitoring in experiments, automation, IoT	High-precision industrial processes, quality control	The DS18B20 is ideal for experimental and educational instrumentation.

### 5.5 Volumetric flow

The volumetric flow in the absorption columns was instrumented and controlled to operate the system continuously. A solenoid valve and a voltage regulator module were employed to regulate the volumetric flow, while a digital Hall-effect flowmeter was utilized for flow measurement. The volumetric flow rate for operating the absorption columns depends on the voltage supplied to the solenoid valve. Subsection 2.2 shows the electrical connections of the elements to the instrument and control the volumetric flow variable.

### 5.6 Stream select

**Fig. 10:**
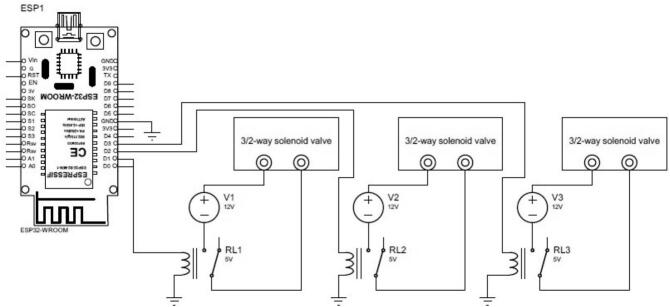
Electrical connection of the 3/2-way solenoid valves.

The post-combustion CO_2_ capture process involves two absorption columns with different capacities. Therefore, three 3/2-way solenoid valves were installed, allowing the configuration of which column to operate during experimental testing. The valves are located at the columns' inlet and outlet streams. Fig. 10 shows the electrical connection diagram of the 3 3/2-way solenoid valves implemented in the system.

### 5.7 Internal wiring

**Fig. 11:**
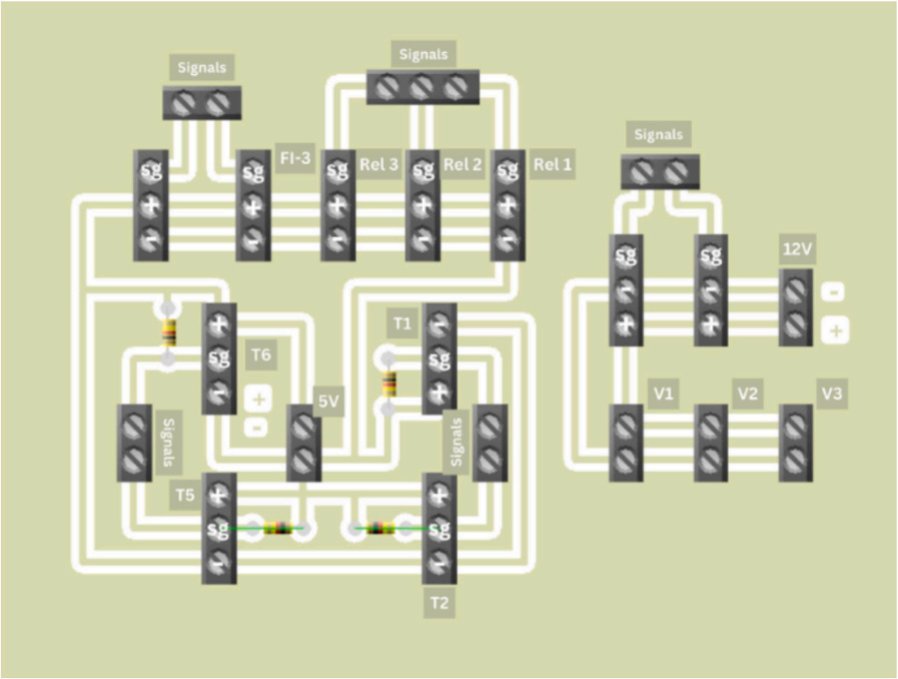
Manufactured design of PCB1. (Nomenclature in Table 2).

Due to the specific work conditions and quantity of instruments, two printed circuit boards (PCBs) were manufactured for the electrical connections for every instrument. The PCB1 board was designed for the absorption columns of the post-combustion system. This PCB, shown in Fig. 11, contains 4 temperature sensors, 1 digital flowmeter, 1 proportional solenoid valve, 1 voltage regulator module, and 2 double relay modules, considering their voltage supply at 12 and 5 V, in addition to the respective signals of each of the elements.

**Fig. 12:**
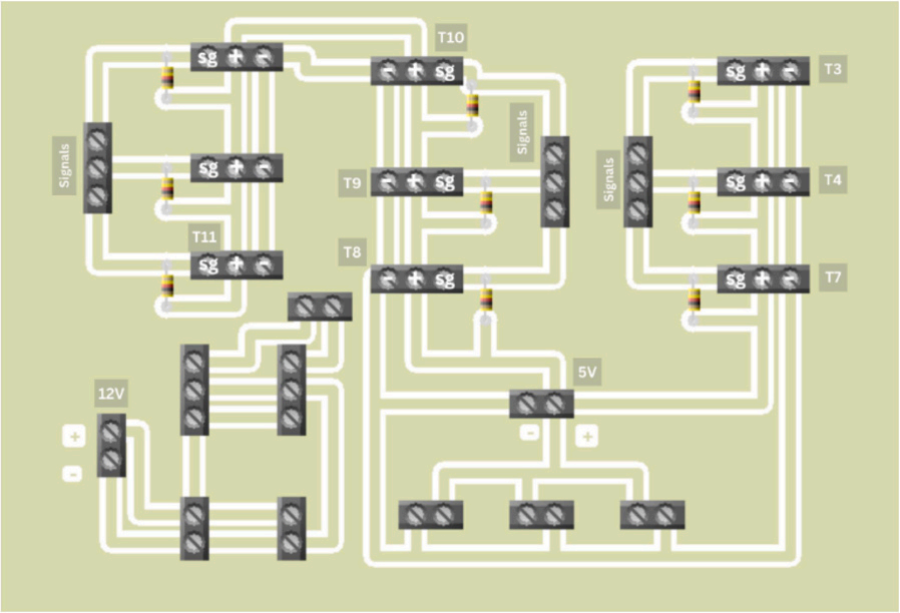
Manufactured design of PCB2. (Nomenclature in Table 2).

The PCB2 board was designed for the desorption column of the post-combustion system. This PCB, shown in Fig. 12, contains 7 temperature sensors, as well as additional connections for future instrumentation.

**Fig. 13:**
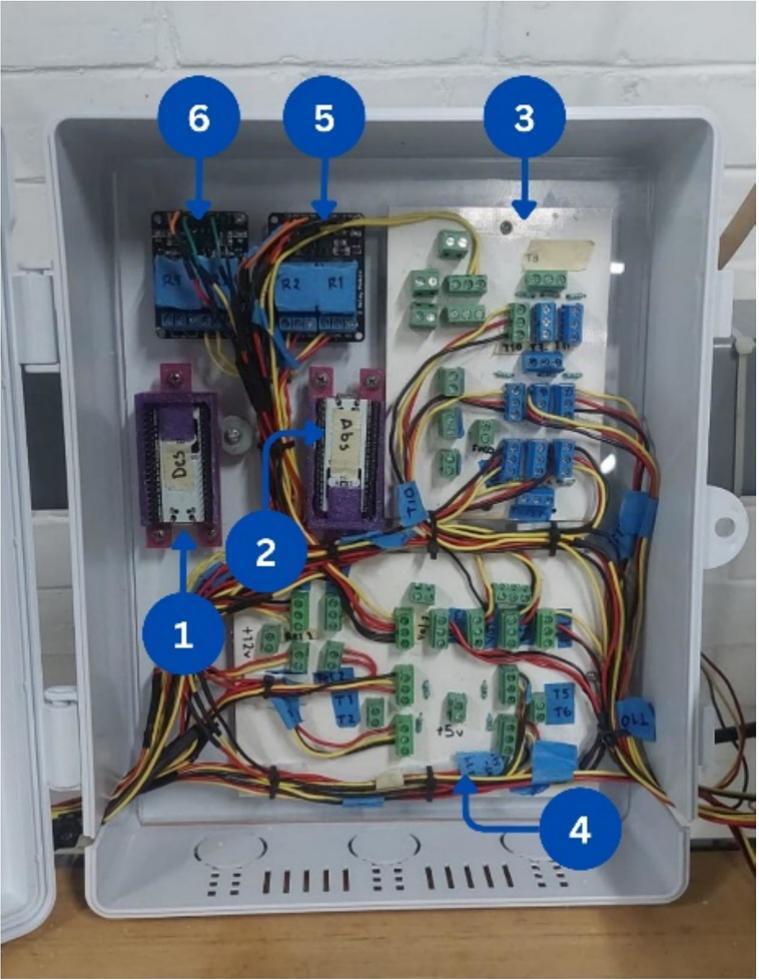
CO_2_-CP system control cabinet.

The PCBs were manufactured and assembled, integrating the modules and connections necessary for the instrumentation and control of the process. Fig. 13 shows the electrical control panel for the process. The panel has two ESP32 cards (1–2), one assigned to the absorption column sensors (3), and the other to the desorption column sensors (4).

## 6 Operation instructions

Consult the repository where the CO_2_-CP System operating manual was added [1].

## 7 Validation and characterization

In this section, the correct operation of the instrumentation and control of the process variables was validated. Table 7 shows the operating conditions for the post-combustion CO_2_ capture process at the laboratory scale.

**Table 7:** General operating parameters.

Variable	Parameters
System pressure	0.8 atm
Steam generator pressure	4.5 atm
Ambient temperature	20 °C
Relative humidity	50 – 65%
Gas flow rate (air)	2 – 10 L/min
Gas flow rate (CO_2_)	0.1 – 1 L/min
Liquid stream feed flow	10 – 40 ml/min
MEA concentration	10 – 30 %w/w
CO_2_ concentration at the gas stream inlet	5 – 12.5 %v/v

### 7.1 Temperature variable

**Fig. 14:**
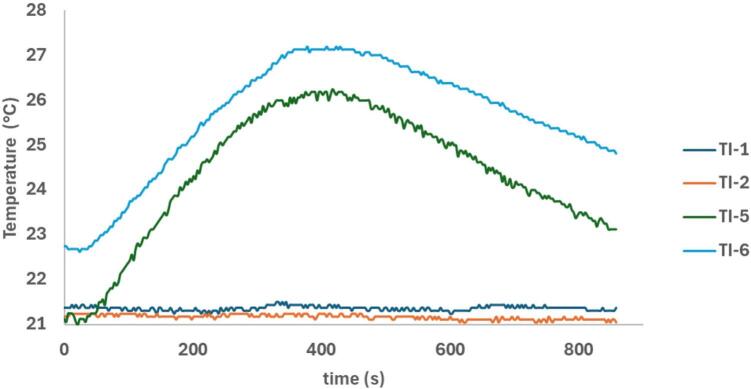
Temperature sensors in the smaller absorption column (0.25 m packed area).

The correct operation of sensors TI-1 − TI-6 was validated through experimental saturation tests, which consist of measuring the temperature of the process streams at the inlets and outlets of the absorption columns. Fig. 14 shows the temperatures acquired during the absorption tests performed in the smaller column, the TI-1 and TI-2 are kept at ambient temperature since they are located at the inlets of the column, sensors TI-5 and TI-6 measured the heat released during absorption, thereby confirming the exothermic reaction. This behavior was reflected by an increase in the temperature of the absorbent solution as it reacted with the CO_2_ flowing in countercurrent. To verify that this thermal behavior was independent of the column size, the same experimental procedure was subsequently applied to the larger absorption column. Similarly, experimental tests were carried out on the large absorption column, sensors TI-3 and TI-4 exhibited temperature profiles comparable to those recorded by the outlet sensors of the smaller column, demonstrating the repeatability and consistency of the absorption process under different operating configurations.

**Fig. 15:**
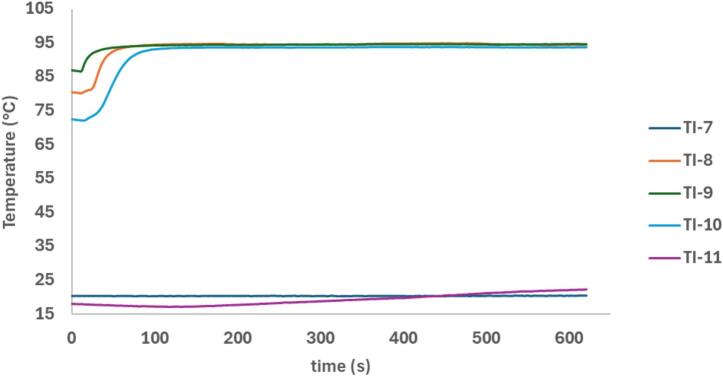
Temperature sensors in the desorption column.

After validating the instrumentation of the absorption stage, the performance of the sensors installed in the regeneration stage was evaluated. The validation TI-7 − TI-11 sensors was carried out using an experimental desorption test to regenerate the absorbent solution used in the CO_2_ capture process. Saturated steam generated by the steam generator was employed as the heat source. The temperature profiles obtained during the desorption process confirmed the proper operation of the monitoring system. Fig. 15 shows the constant temperature of the saturated absorbent solution environment. Sensor T11 registers a slight increase in the cooling fluid temperature because the fluid is recirculating in the same storage tank. Likewise, sensors T8, T9, and T10 show an increase in temperature until reaching the steady state, corresponding to the saturation temperature of the steam used in the process.

### 7.2 Volumetric flow variable

**Fig. 16:**
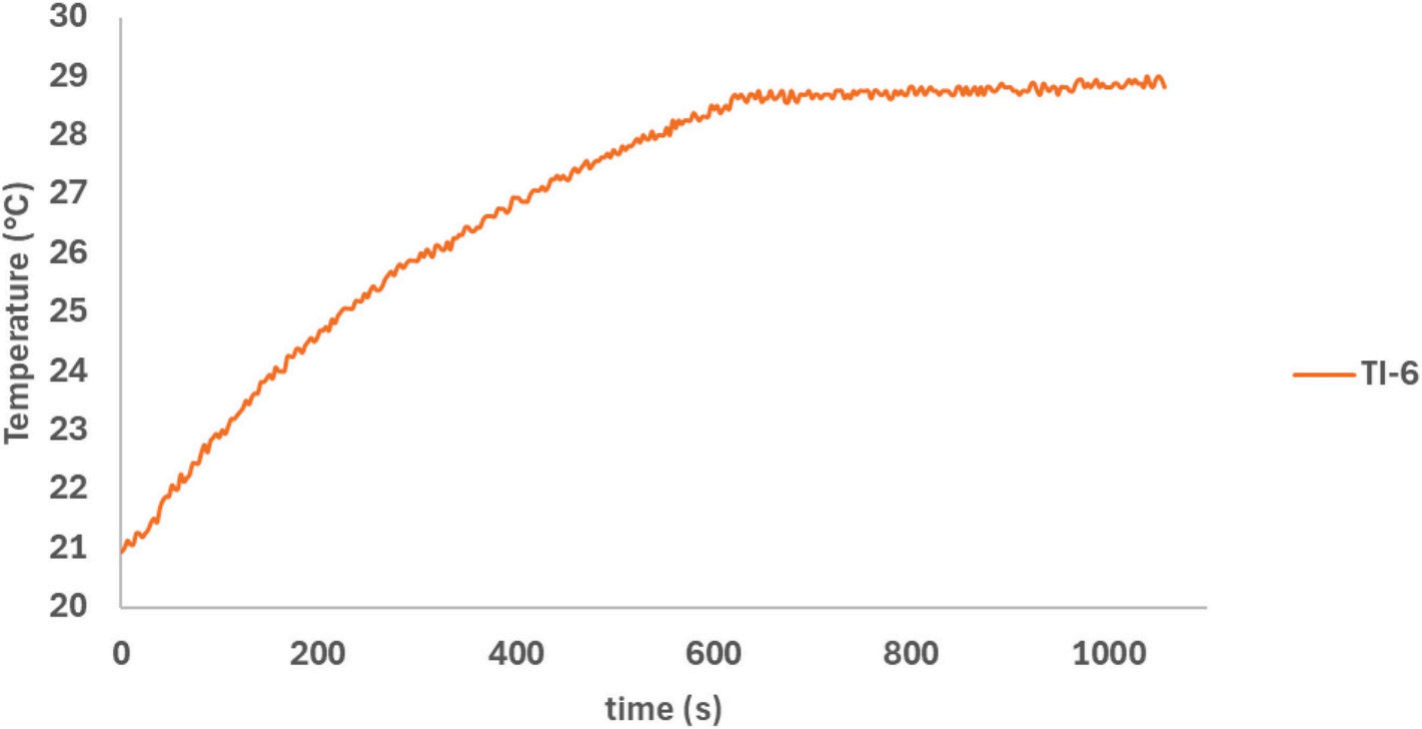
Validation of the continuous process (EV-1, FI-3).

The volumetric flow of the absorbent solution (MEA-H_2_O) was instrumented and controlled. For this purpose, an EV-1 solenoid valve was used; the opening and closing process was controlled by a voltage regulator module that supplied the necessary voltage. In addition, a FI-3 digital Hall effect flowmeter and a TI-6 temperature sensor were integrated at the outlet of the saturated solution. The countercurrent contact between the absorbent solution and the CO_2_ stream inside the absorption column promotes an exothermic reaction, demonstrating an increase in the temperature of solution [18]. Fig. 16 shows the temperature profile of the absorbent solution as a function of time for a 10 % MEA concentration.

The implementation of the temperature sensors implemented in the CO_2_-CP system allows the temperature variable to be monitored in real time until it reaches a steady state in the various tests performed.

In addition to temperature monitoring, maintaining an appropriate flow balance is essential for ensuring stable process operation. On the other hand, the implementation of the flow control system ensures compliance with the continuity principle, which states that the volumetric flow rate entering the absorption column equals the volumetric flow rate leaving the system under steady-state conditions.

From a hardware integration perspective, the developed electronic architecture also contributes to improving the overall reliability of the data acquisition system. The incorporation of dedicated printed circuit boards (PCB) improves the organization of measurement and control devices, enhances signal stability, and reduces electrical noise, thereby enhancing the reliability and quality of the acquired experimental data.

## 8 Conclusions

**Fig. 17:**
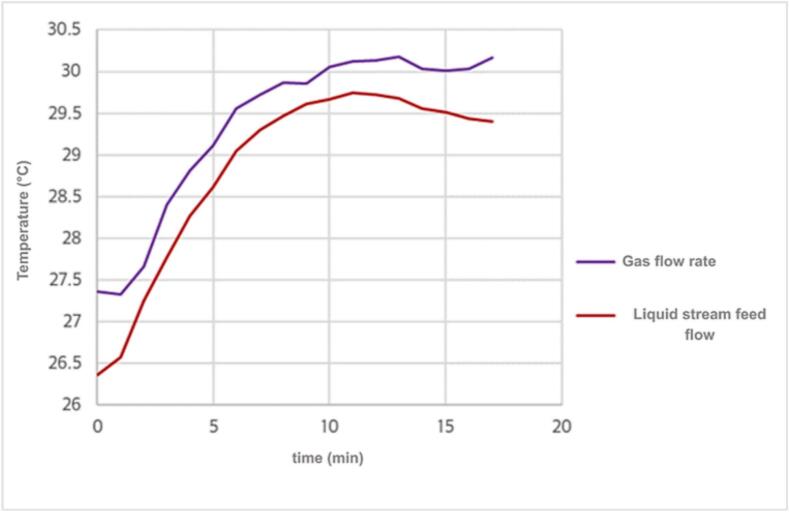
Data acquisition before instrumentation and control.

**Fig. 18:**
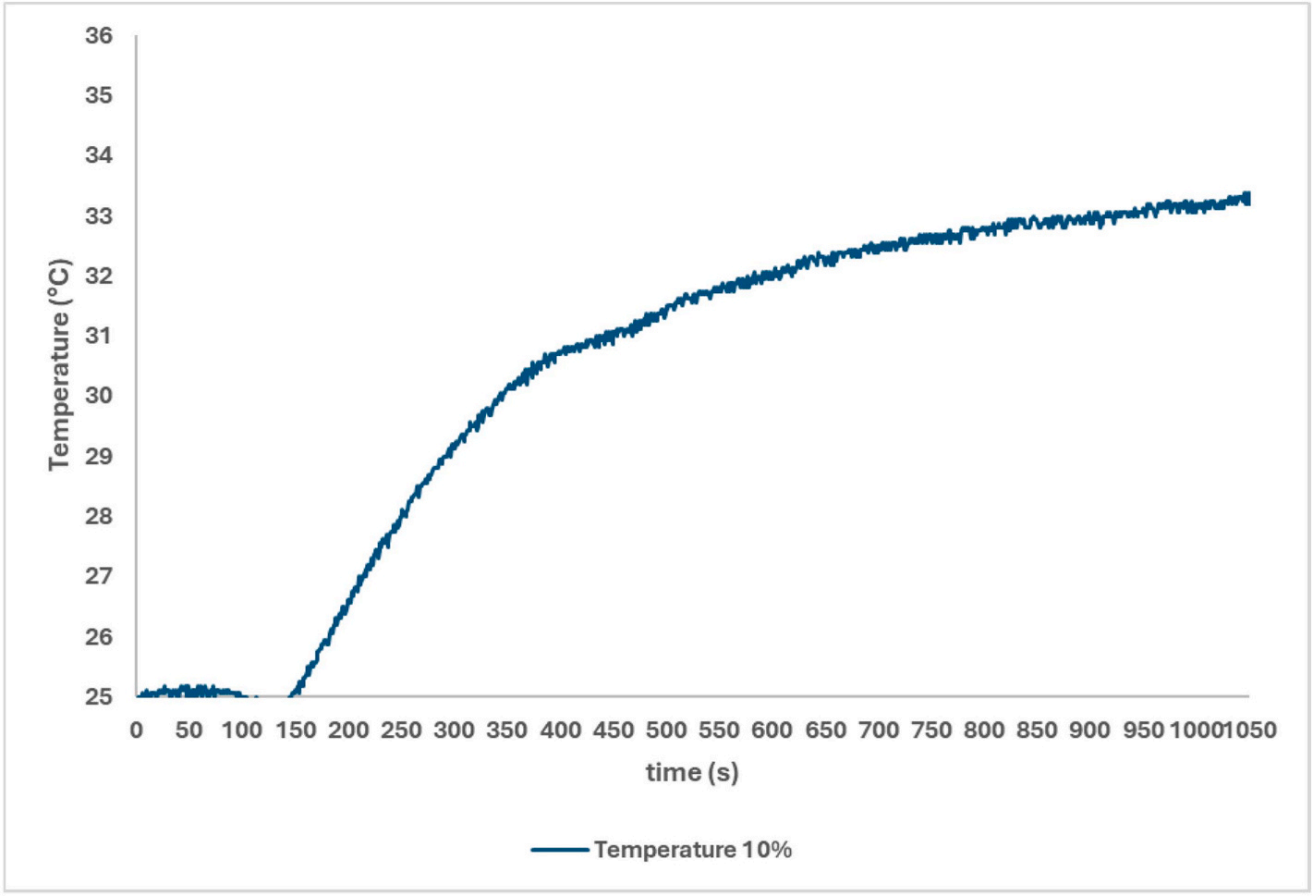
Data acquisition before instrumentation and control.

**Fig. 19:**
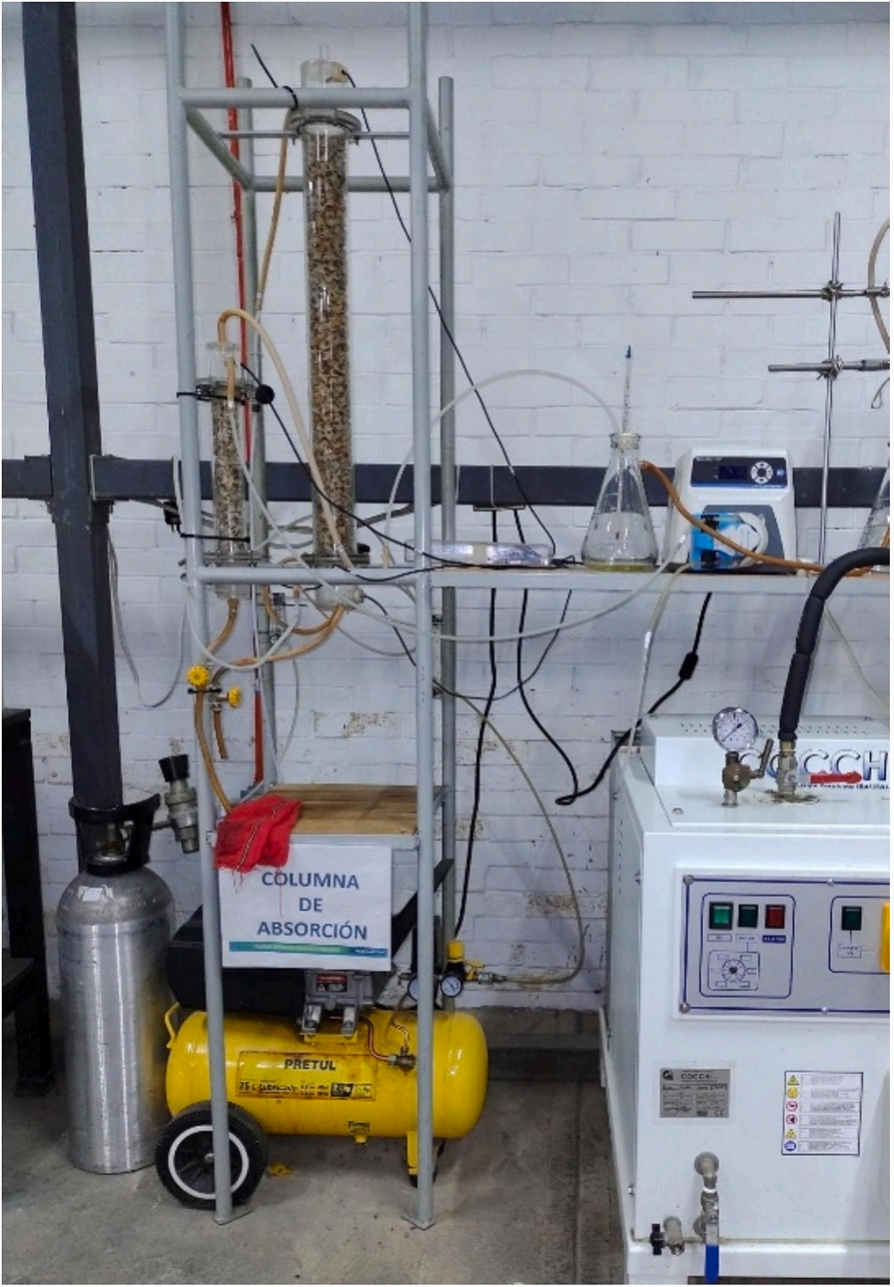
System before implementation of the instrumentation and control.

**Fig. 20:**
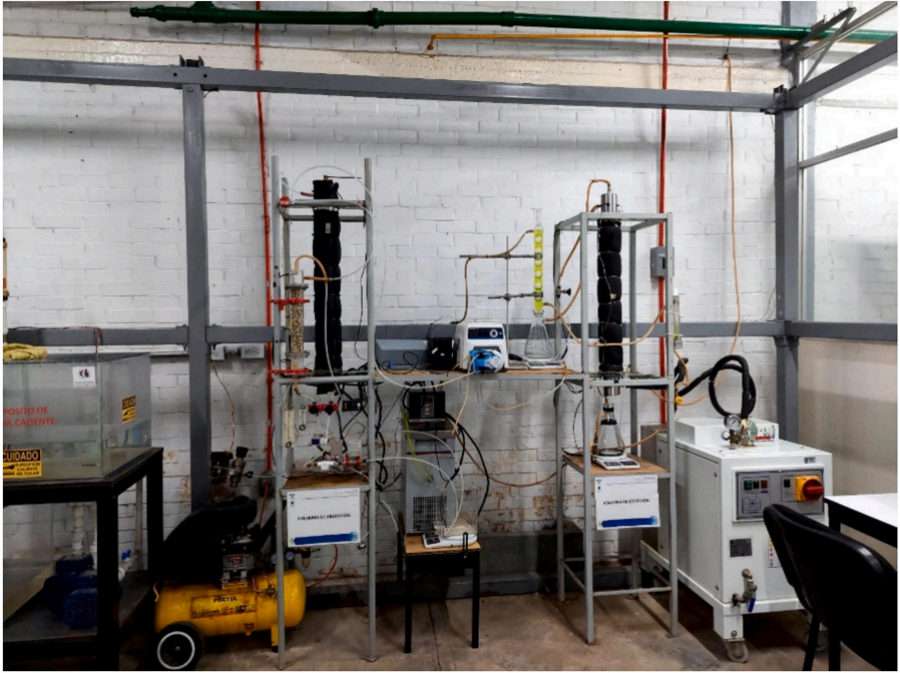
System after implementation of the instrumentation and control.

With respect to the previous reports LOU configuration, Fig. 17, Fig. 18 compare the system’s behavior before and after the implementation of the proposed instrumentation and control platform. This comparison clearly illustrates the improvements achieved in data acquisition and process monitoring during experimental tests conducted under different operating conditions.Fig. 19Fig. 20.

Real-time temperature monitoring enables the user to verify that the CO_2_ captures process is operating correctly, while simultaneously acquiring reliable experimental data for subsequent analysis. This capability not only facilitates process supervision but also enables early identification of abnormal operating conditions, thereby improving the safety and reliability of the experimental system.

In addition to temperature monitoring, stable flow regulation is essential to ensure continuous operation of the absorption process. Consequently, the implementation of volumetric flow control prevents the absorption column from either flooding or running dry, significantly reducing operator intervention during experimental testing.

To further improve the usability of the proposed platform, a dedicated graphical interface was developed for real time visualization and process management. The implementation of this low-cost data acquisition system enables users to continuously monitor operating variables during experiments. In addition to meeting system requirements, such as installing sensors at strategic locations, the flow control system enables the operator to select the absorption column based on the experimental conditions being investigated.

The overall impact of the proposed instrumentation strategy is illustrated in figures 19 and 20, which present the CO_2_-CP system before and after the implementation of the instrumentation and control platform.

To ensure the reliability of the acquired measurements, all temperature sensors were calibrated prior to the experimental evaluation. The calibration results show agreement between the corrected and reference temperatures for all evaluated sensors. The coefficient of determination (R^2^) ranged from 0.99722 to 0.99989 units, indicating a linear correlation and confirming the suitability of the proposed calibration equations. The Mean Squared Relative Error (MSRE) values remained below 0.0009 °C, while the Mean Absolute Error (MAE) ranged from 0.145 °C to 0.869 °C. Likewise, the Maximum Absolute Error (Max AE) was lower than 1.38 °C for all sensors.

Overall, the statistical indicator confirms that the proposed calibration methodology significantly improved the accuracy and reliability of the temperature measurements, providing high quality experimental data for subsequent thermal analysis and process evaluation. The corrected accuracy (CA) remained above 94% for all calibrated sensors.

## Ethics statements

No human or animal studies were conducted in the design of this work.

## CRediT authorship contribution statement

**María Fernanda Silva León:** Writing – original draft, Methodology, Conceptualization. **Erick Omar Castañeda Magadán:** Writing – review & editing, Writing – original draft, Supervision, Investigation, Data curation. **Miriam Navarrete Procopio:** Writing – review & editing, Writing – original draft, Validation. **Víctor Manuel Zezatti Flores:** Writing – review & editing, Writing – original draft, Visualization, Supervision, Investigation. **Ángel Tlatelpa Becerro:** Supervision.

## Declaration of competing interest

The authors declare that they have no known competing financial interests or personal relationships that could have appeared to influence the work reported in this paper.
